# Systemic Effects of Anabolic-Androgenic Steroid Abuse: A Case in Primary Care

**DOI:** 10.7759/cureus.93589

**Published:** 2025-09-30

**Authors:** Margarida de Carvalho Vilarinho, Joana Cernadas, Miguel Marques Ferreira

**Affiliations:** 1 General Practice, Unidade Local de Saúde de São José, Lisbon, PRT; 2 Family Medicine, Unidade Local de Saúde de São José, Lisbon, PRT

**Keywords:** anabolic-androgenic steroids, bodybuilding, family medicine, gynecomastia, hepatotoxicity, hypogonadism, muscle atrophy, primary care, total testosterone

## Abstract

Anabolic-androgenic steroid (AAS) abuse is a growing public health concern due to its wide-ranging physical and psychological effects. This case report highlights the multisystemic consequences of prolonged AAS use in a 30-year-old male presenting initially with a minor respiratory infection during a consultation in his healthcare center. Despite his primary complaint, upon physical examination, muscle hypertrophy, bilateral gynecomastia, and suspected testicular atrophy were observed, prompting further investigation. Laboratory findings showed high levels of testosterone, suppressed gonadotropins, elevated estradiol, dyslipidemia, and liver enzyme elevation, consistent with chronic AAS abuse. Imaging confirmed gynecomastia, and the patient was diagnosed with hypertension, likely secondary to AAS use. Despite counseling on the potential short- and long-term health risks, the patient chose to continue AAS use. Ongoing follow-up in primary care, however, made it possible for the patient to return to care after one year, when he presented with depressive symptoms and body image dissatisfaction following steroid cessation, demonstrating the critical role of family physicians in early detection, patient education, and motivational support. This case shows the importance of a thorough approach in identifying AAS abuse and managing its systemic effects to mitigate potentially perilous health outcomes.

## Introduction

Performance-enhancing drugs are agents used to enhance athletic performance and physical appearance, giving a leaner and more muscular appearance [1]. Many believe that only athletes from competitive sports use drugs to improve their performance. However, the majority of individuals using these medications are recreational athletes such as weightlifters and bodybuilders, who use them as a strategy to increase muscle mass, improve performance, and enhance physical attractiveness [1].

The most frequently used class of performance-enhancing drugs is anabolic-androgenic steroids (AAS), which decrease body fat mass and increase lean body mass [1]. Nonmedical androgenic steroids are a serious global public health problem: In a comprehensive meta-analysis of 187 studies, Sagoe et al. reported a global lifetime prevalence of 3.3% (95% confidence interval (CI: 2.8-3.8; reported heterogeneity (I²) = 99.7; p < 0.001). However, the prevalence was substantially higher among males (6.4%, 95% CI: 5.3-7.7; I² = 99.2; p < 0.001) compared to females (1.6%, 95% CI: 1.3-1.9; I² = 96.8; p < 0.001), a difference that was statistically significant (Cochran’s Q for between-study heterogeneity (Qbet) = 100.1; p < 0.001) [1].

High doses of AAS are associated with a wide spectrum of adverse effects across multiple organ systems. Endocrine manifestations result from AAS suppression of the hypothalamic-pituitary-gonadal (HPG) axis via negative feedback, with reduction of gonadotropin-releasing hormone (GnRH), luteinizing hormone (LH), and follicle-stimulating hormone (FSH) secretion. In males, well-documented endocrine complications include gynecomastia, testicular atrophy, diminished spermatogenesis, infertility, and androgenic alopecia. In females, common endocrine manifestations include hirsutism, clitoromegaly, acne, androgenic alopecia, and deepening of the voice, also attributable to androgen excess [2-4].

In both sexes, observational studies consistently report increased risks of hypertension, dyslipidemia (decreased serum high-density lipoprotein (HDL) cholesterol and increased low-density lipoprotein (LDL) cholesterol concentrations), and premature myocardial infarction among AAS users, while mechanistic evidence supports steroid-induced cardiac hypertrophy, prothrombotic alterations, and vascular dysfunction [5-11]. Hepatotoxicity is another major concern: case series and observational data describe cholestasis, peliosis hepatis, and both benign and malignant hepatic tumors, although less likely. AAS drug-induced liver injury frequently leads to elevated serum liver enzymes [12]. AAS use also has neuropsychiatric disturbances such as aggressive behavior, mood disorders, depression, and body dysmorphia - frequently reported in observational studies. With respect to hormone-sensitive cancers, current evidence remains limited to observational reports and experimental models, and causality is uncertain [3,13,14]. Despite these risks, many users underestimate the potential harm, making early detection and counseling essential.

This case report aims to describe clinical features, investigations, and primary care management of suspected AAS-associated complications in a young male and emphasize the essential role of a family physician in the early detection of such cases.

This case report was prepared in accordance with the CARE Case Report Guidelines, and written informed consent was obtained from the patient for publication of clinical details. Approval from a research ethics committee was not required because it does not involve experimental intervention.

## Case presentation

A 30-year-old male, originally from Brazil, with no known past medical history, presented to an urgent medical appointment in his healthcare center with symptoms of a respiratory infection, such as rhinorrhea, sneezing, and sore throat, in December 2023. During physical examination, aside from findings consistent with an upper respiratory tract infection, there was an evident hypertrophy of all muscle groups and bilateral gynecomastia (grade II A according to Simon's gynecomastia classification system) and a firm, non-tender, well-circumscribed, mobile nodule measuring approximately 1 x 1 cm in the left peri-areolar region, with no associated skin or nipple changes and no palpable axillary lymphadenopathy.

Following the management of the presenting complaint that took him to the health care center, further medical inquiries revealed a history of anabolic steroid abuse for four years, reporting cycles of intramuscular testosterone cypionate for 12 weeks on and 12 weeks off with no concomitant agents being used. The patient practiced bodybuilding on a daily basis and participated in several competitions.

Despite ongoing AAS use, he denied the use of any other daily medications, dietary supplements, or herbal products. He also denied consumption of alcohol, tobacco, or other recreational or illicit substances. Upon further questioning, the patient stated his intention to continue using anabolic steroids. He reported no changes in sexual performance, specifically with regard to libido, ejaculation, or erectile dysfunction, according to the International Index of Erectile Function-5 (IIEF-5) scale (24 points out of 25 in total).

This information prompted further physical examination of the patient: his weight was 89 kg, height was 172 cm, body mass index (BMI) of 30.1 kg/m^2^ (kilograms per square meter), the testicles appeared to be reduced in size during manual palpation (there was no orchidometer available to quantify), and elevated blood pressure was measured during the consultation: 150/85 mmHg (millimeters of mercury).

These initial findings suggest suspected systemic consequences of anabolic-androgenic steroid abuse: gynecomastia and testicular atrophy reflect endocrine disruption through suppression of the HPG axis; hypertension raises concern for cardiovascular risk; and generalized muscular hypertrophy is a physical marker of chronic exposure.

**Figure 1:**
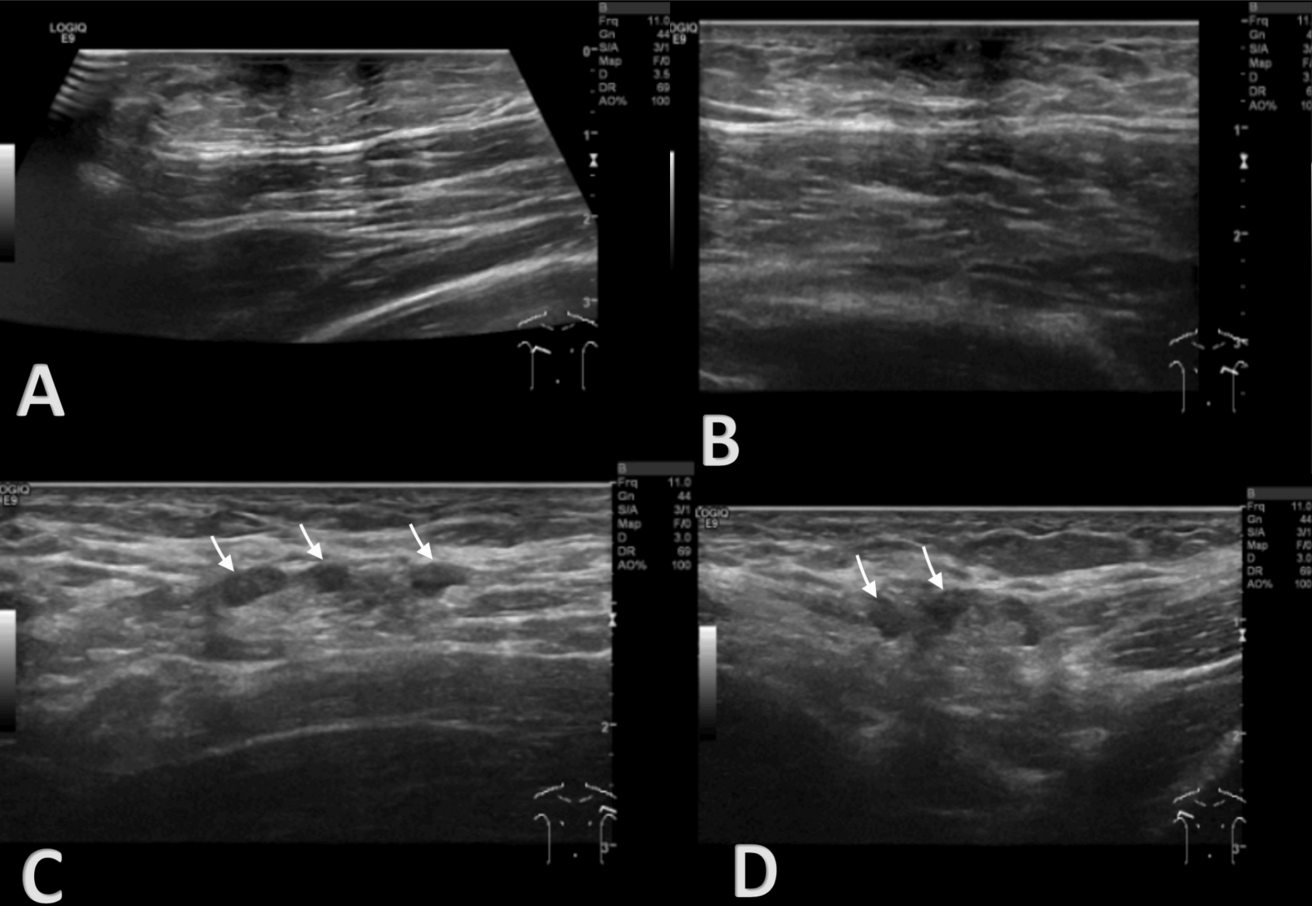
Breast ultrasound A: right breast; B: left breast; C: left axilla; D: right axilla Findings show mild bilateral dendritic gynecomastia (A, B) and bilateral reactive axillary lymphadenopathy with preserved fatty hilum (white arrows in C and D).

**Figure 2:**
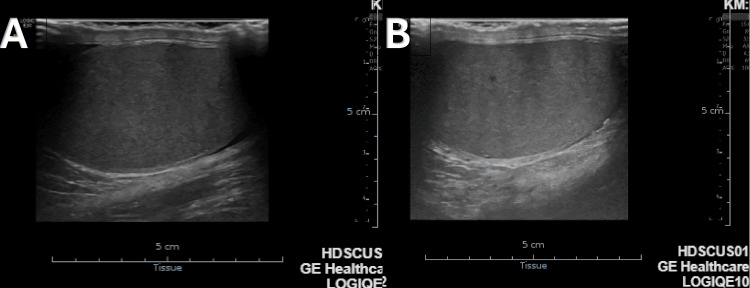
Testicular ultrasound A: right testicle; B: left testicle. Right and left testicle volume 16 cubic centimeter (cm^3^).

To further investigate the gynecomastia and evaluate suspected AAS-associated effects, a thorough evaluation was requested, including laboratory testing (Table 1), breast ultrasound (Figure 1), testicular ultrasound (Figure 2), and home blood pressure monitoring (HBPM), which the patient showed at a follow-up consultation in March 2024.

**Table 1:** Blood analysis Laboratory results before (2019) and after (2024) reported anabolic-androgenic steroid (AAS) use. x10^6^/mm^3^: millions of cells per cubic millimeter; g/dL: grams per deciliter; %: percent; mg/dL: milligrams per deciliter; ng/dL: nanograms per deciliter; pg/mL: picograms per milliliter; µg/dL: micrograms per deciliter; ng/mL: nanograms per milliliter; mU/mL: milliunits per milliliter; nmol/L: nanomoles per liter; mUI/L: milli-international units per liter; mUl/mL: milli-international units per milliliter; U/L: units per liter

Laboratory assessment	12/05/2019	14/02/2024	Reference range	Units
Erythrocytes	-	5.22	4.7-6.1	x10^6^/mm^3^
Hemoglobin	-	16.1	14-18	g/dL
Hematocrit	-	45.5	40-54	%
Total cholesterol	185	307	<190	mg/dL
Triglycerides	75	115	<150	mg/dL
HDL cholesterol	51	27	>60	mg/dL
LDL cholesterol	119	257	-	mg/dL
Glucose	87	73	70-110	mg/dL
Creatinine	0.85	1.02	0.5-1.1	mg/dL
Total testosterone	-	3606.4	241-827	ng/dL
Free testosterone	-	149.2	7.72-31.4	pg/mL
DHEA-S (dehydroepiandrosterone sulfate)	-	157	99-340	µg/dL
Alpha fetoprotein (AFP)	-	3.7	<7	ng/mL
Prolactin (PRL)	-	7.8	2-18	ng/mL
Estradiol 17 beta (E2)	-	123.7	<32	pg/mL
Follicle-stimulating hormone (FSH)	-	0.3	1.4-18.1	mU/mL
Luteinizing hormone (LH)	-	0.07	1.5-9.3	mU/mL
Sex hormone binding globulin (SHBG)	-	3.81	10-57	nmol/L
Thyroid-stimulating hormone (TSH)	-	1.78	0.4-4.2	mUI/L
Free T4 (thyroxine)	-	1.54	0.7-1.8	ng/dL
Human chorionic gonadotropin (hCG)	-	0.2	<2	mUl/mL
Aspartate aminotransferase (AST)	-	76	<34	U/L
Alanine aminotransferase (ALT)	-	79	<49	U/L
Gamma-glutamyl transferase (GGT)	-	18	<38	U/L
Alkaline phosphatase (ALP)	-	35	30-130	U/L

Laboratory testing (Table 1) from May 2019, prior to reported AAS exposure, showed no relevant abnormalities. In February 2024, results demonstrated markedly elevated total testosterone (3,606 nanograms per deciliter (ng/dL)) and free testosterone (149 picograms per milliliter (pg/mL) - method of assessment not specified), with suppressed gonadotropins (FSH 0.3 milliunits per milliliter (mU/mL), LH 0.07 milli-international units per milliliter (mU/mL)), a pattern supportive of exogenous androgen administration. Estradiol was also elevated (124 picograms per milliliter (pg/mL)). Lipid profile showed severe hypercholesterolemia with very high LDL cholesterol (257 milligrams per deciliter (mg/dL)) and low HDL (27 milligrams per deciliter (mg/dL)).

Liver enzymes were mildly elevated (aspartate aminotransferase (AST) of 76 units per liter (U/L), alanine aminotransferase (ALT) of 79 units per liter (U/L), normal Gamma-glutamyl transferase (GGT)), consistent with a hepatocellular pattern (R-ratio for liver injury 5,3), although alternative causes should be considered. Absolute cardiovascular risk assessment is limited at age 30, but dyslipidemia warrants follow-up after cessation. Red blood cell count was within reference values, with no evidence of erythrocytosis. Repeating endocrine and metabolic testing after six to eight weeks off-cycle would help document recovery or persistent hypogonadism and metabolic derangements.

Regarding breast ultrasound (Figure 1), findings were consistent with mild bilateral dendritic gynecomastia (A: right breast; B: left breast). No suspicious nodular lesions were identified, particularly at the outer border of the left pectoralis major muscle or in the left peri-areolar region (B), where the patient had previously palpated a nodule that had since completely resolved. In both axillae, lymph nodes appeared mildly hypertrophic but symmetrical, with preserved fatty hilum (C: left axilla; D: right axilla), findings most consistent with reactive changes, possibly suspected AAS-associated. These results were scored by Breast Imaging Reporting and Data System (BI-RADS) in category 2 - benign findings.

Concerning testicular ultrasound (Figure 2), testicles demonstrated preserved size (unlike what was evaluated on manual palpation) and symmetrical morphology; testicular echotexture was homogeneous, epididymides showed normal ultrasound characteristics, and there was no hydrocele nor varicocele identified.

HBPM revealed values consistent with grade I hypertension for the patient’s age. Hypertension was considered likely secondary to anabolic-androgenic steroid exposure, though other secondary causes - including renal, endocrine, and medication-related etiologies - were evaluated and excluded based on anamnesis, laboratory, and imaging assessments. Antihypertensive therapy with olmesartan 10 mg (milligrams) once daily, an angiotensin II receptor blocker appropriate for young adults with uncomplicated hypertension, was recommended to achieve a target systolic blood pressure of 120-129 mmHg and a diastolic blood pressure of < 80 mmHg; however, the patient declined pharmacologic treatment. The refusal was documented, and the patient was advised to continue HBPM, adopt lifestyle measures, and seek urgent medical care for severe elevations or symptoms. Repeat assessment is planned six to eight weeks after steroid cessation to evaluate persistence of hypertension. Furthermore, he was informed of the possible consequences associated with substance abuse, such as secondary hypertension, dyslipidemia, infertility, hepatic toxicity, gynecomastia, and the potential risk of testicular atrophy and developing cancers. Following the results presented, a referral to endocrinology was requested, and despite the advice and information provided, the patient still demonstrated the desire to continue using AAS and declined to continue treatment; therefore, he was discharged from hospital specialty care.

The patient continued follow-up with his family doctor, where brief interventions to support discontinuation of anabolic steroid use were performed. In February 2025, he presented with low mood and dissatisfaction regarding persistent gynecomastia. Screening with the Patient Health Questionnaire-9 (PHQ-9) indicated mild depressive symptoms. He reported discontinuing anabolic steroid use two weeks earlier and expressed motivation to improve his health. The patient was referred to a psychology appointment, and follow-up was scheduled to monitor mood, adherence to discontinuation, and risk of relapse.

This case highlights the suspected systemic impact of AAS abuse, involving endocrine, cardiovascular, hepatic, and psychological domains, with complications detected in primary care during evaluation for an unrelated complaint. Continuous follow-up in primary care enabled early recognition of these complications, monitoring of mental health, and timely referral, underscoring the pivotal role of family physicians in identifying early suspected signs of AAS misuse and managing substance-related harms.

**Figure 3:**
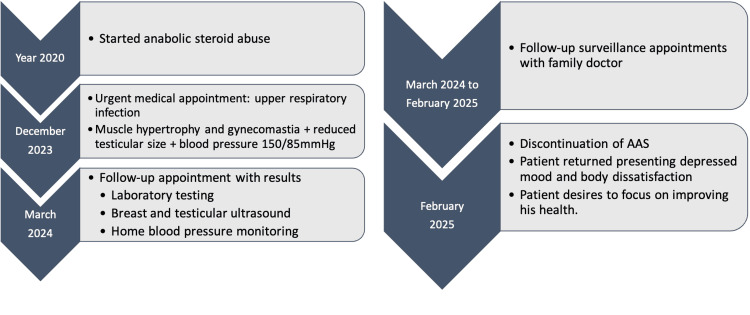
Timeline of key clinical events Schematic representation of the patient’s clinical course, including initial symptoms, laboratory and imaging findings, and follow-up. AAS: Anabolic-androgenic steroid

Schematic representation of the patient’s clinical course, including initial symptoms, laboratory and imaging findings, and follow-up, is shown in Figure 3.

## Discussion

This case illustrates the broad spectrum of suspected AAS-associated health effects and the essential role of a family physician in their detection and follow-up. Although the patient initially presented with a minor upper respiratory infection, careful history-taking and physical examination revealed findings consistent with long-term performance-enhancing drug use, including muscle hypertrophy and gynecomastia. Gynecomastia in AAS users is understood to result from aromatization of testosterone to estradiol, a mechanism well documented in the literature [15]. Steroid-associated gynecomastia has been reported to constitute up to 10-25% of cases and may persist even after discontinuation [15].

The temporal evolution of his results is notable: laboratory testing in 2019, prior to reported AAS use, showed no relevant abnormalities, whereas in 2024, after several years of intramuscular testosterone cypionate use, he demonstrated markedly elevated testosterone, suppressed gonadotropins, and increased estradiol. This pattern is physiologically consistent with exogenous androgen exposure and suspected secondary hypogonadism, though not pathognomonic. Suppression of the HPG axis by supraphysiologic androgens is well described, and in some individuals, hypogonadism may persist months to years after cessation (anabolic steroid-induced hypogonadism) [16].

In addition, cardiovascular and metabolic abnormalities were documented, including severe dyslipidemia, mild hepatocellular enzyme elevation, and grade I hypertension. Each of these is suspected to be AAS-associated based on prior literature, although alternative etiologies cannot be excluded, and repeat testing after AAS discontinuation is required for confirmation. AAS use has consistently been associated with adverse lipid changes, hypertension, and cardiac remodeling [17]. The observed hepatic enzyme elevation showed a hepatocellular pattern, which may be compatible with subtle AAS-related hepatotoxicity reported in reviews, although other common causes must be excluded, such as alcohol, viral hepatitis, or non-alcoholic fatty liver disease [12].

Psychological manifestations emerged following self-reported cessation, with low mood and body image dissatisfaction. These symptoms may reflect suspected AAS withdrawal and body dysmorphic concerns, but they are nonspecific and should be systematically assessed using standardized tools, with appropriate mental health referral and follow-up. Withdrawal-related psychiatric symptoms, including depression, anhedonia, irritability, and decreased libido, have been documented in former AAS users [18]. This case emphasizes the challenges in counseling individuals who are reluctant to discontinue AAS despite being informed of potential health risks. Persistent engagement, motivational interviewing, and patient-centered approaches remain crucial, as demonstrated by the patient’s eventual return to care after initial resistance. Recent reviews highlight the importance of harm reduction and motivational interviewing in managing patients who use AAS [19]. For primary care physicians, maintaining a high index of suspicion for AAS use- particularly among young men involved in weightlifting with suspected signs- is essential to guide appropriate investigations, monitor for multisystem abnormalities, and provide harm-reduction counseling. Therefore, a detailed and nonjudgmental approach is key to recognize potentially perilous habits, direct pertinent investigations, and offer advice to mitigate adverse complications.

Key Points for Primary Care Practice

Consider AAS use in young men presenting with muscle hypertrophy and gynecomastia. 

Laboratory and clinical clues, including markedly elevated testosterone with suppressed LH/FSH, dyslipidemia, hypertension, and liver enzyme elevations, may indicate suspected AAS-related endocrine and metabolic disturbances but require confirmation with repeat testing after discontinuation.

Primary care physicians are central in providing nonjudgmental counseling, motivational interviewing, and ongoing support for patients reluctant to discontinue AAS while monitoring for multisystem complications.

## Conclusions

This case highlights how AAS use can possibly produce systemic health effects - endocrine, cardiovascular, hepatic, and psychological - that may be recognized in primary care, often when patients present for unrelated complaints. The temporal association between prolonged intramuscular testosterone use and abnormalities such as gynecomastia, dyslipidemia, hepatocellular enzyme elevation, and mood disturbance illustrates the wide range of suspected AAS-related harm.

For family physicians, the case highlights the importance of maintaining clinical suspicion, asking targeted but nonjudgmental questions, and providing longitudinal follow-up in patients with suspicious findings. Beyond early recognition and referral, the physician’s role extends to persistent engagement, motivational interviewing, and harm-reduction counseling, which may create opportunities for change even in patients initially resistant to discontinuation. As AAS use continues to rise globally, particularly among younger non-athlete patients, this case emphasizes the need for primary care providers to remain vigilant, informed, and proactive in detecting complications, supporting safe discontinuation, and mitigating long-term risks. Awareness of AAS misuse, its consequences, and potential future risks is therefore of paramount importance and should be regarded as an essential component of a family physician’s medical knowledge.
